# H19-derived miR-675 contributes to bladder cancer cell proliferation by regulating p53 activation

**DOI:** 10.1007/s13277-015-3779-2

**Published:** 2015-07-22

**Authors:** Changkun Liu, Zhouguang Chen, Jianzheng Fang, Aiming Xu, Wei Zhang, Zengjun Wang

**Affiliations:** 1 State Key Laboratory of Reproductive Medicine and Department of Urology, The First Affiliated Hospital of Nanjing Medical University, Nanjing, China; 2 Department of Urology, Subei Hospital, Yangzhou University, Yangzhou, China; 3 Clinical Center of Reproductive Medicine, First Affiliated Hospital of Nanjing Medical University, Nanjing, China

**Keywords:** Bladder cancer, miR-675, Proliferation, p53, H19

## Abstract

Long noncoding RNA 19 (H19) has been shown to promote bladder cancer cell proliferation and metastasis. However, little is known about how miR-675, mature product of H19, contributes to bladder cancer cell proliferation. In this study, we first evaluated the expression of miR-675 in bladder cancer tissues by quantitative real-time PCR (qRT-PCR) and defined its biological functions by flow cytometry and Western blotting. We found that miR-675 expression levels were remarkably increased in bladder cancer tissues as compared with adjacent noncancerous tissues or normal bladder tissue from health donors; moreover, enhanced miR-675 expression was also observed in bladder cancer cell lines. Ectopic expression of H19 significantly increased bladder cancer cell proliferation and miR-675 expression in vitro. Furthermore, overexpression of miR-675 promoted bladder cancer cell proliferation, while suppression of miR-675 induced G1 phase cell cycle arrest and promoted cell apoptosis. Western blotting analysis further identified that miR-675 inhibited p53 activation, decreased the ratio of Bax/Bcl-2 and cyclin D1 expression in bladder cancer cells; those effects may result in the abnormal proliferation of bladder cancer cells. In conclusion, abnormal enhanced miR-675 expression increases bladder cancer growth by regulating p53 activation, and thus may be helpful in the development of effective treatment strategies for bladder cancer.

## Introduction

Human bladder cancer was the ninth most common cancer with an estimated 429,793 new cases and 165,068 deaths registered in 2012 worldwide [1]. It was three times more common in men compared with women and ranks sixth among cancers in men [2]. Bladder cancer comprises distinct histopathological patterns and clinical behaviors and thus making serious obstruction in the controlling of bladder cancer [3, 4]. The most common histological type of bladder cancer is urothelial carcinoma, which recurs frequently and the prognosis is generally over 85 % survival at 5 years, rarely accompany with muscle invasion, whereas muscle-invasive bladder cancer frequently progresses to distant life-threatening metastases, with survival rate only about 6 % [5–8]. Therefore, understanding the molecular mechanisms involved in bladder cancer growth is critical to the development of identifying new therapeutic targets for bladder cancer.

Emerging evidence showed that long noncoding RNA 19 (H19) possesses oncogenic properties and is the key regulator in carcinogenesis and metastasis of bladder cancer [9–14]. Upregulated H19 promoted the proliferation and metastasis of bladder cancer cells [10, 9]. Furthermore, H19 RNA level was greatly enhanced in tumor of human bladder cancer cells formed in nude mice and in patients with bladder cancer [11, 12]; it also could serve as a prognostic tumor marker for the early recurrence of bladder cancer [14, 13].

The molecular mechanisms are involved in H19-induced tumor cell proliferation including two aspect: (1) Berteaux et al. demonstrated that H19 RNA was actively linked to E2F1 and thus promote cell cycle progression of breast cancer cells [15]. Furthermore, H19 also has been found to promote cancer cell proliferation by directly inactivating tumor suppressor p53 and increasing ID2 expression [9, 16]. (2) H19 functioned in an indirect way to promote tumor cell proliferation. As the mature product of H19, H19-derived miR-675 regulated tumor cell proliferation through downregulation of its targets, tumor suppressor retinoblastoma (RB) [17], and Runt-related transcription factor 1 (RUNX1) [18]. Particularly, RUNX1 stimulates tumor suppressor p53 protein in response to DNA damage [19–21].

The H19/miR-675 signaling axis has been found to promote progression of different cancers, including colorectal cancer [17], gastric cancer [18], glioma [22], and prostate cancer [23]. However, little is known about whether H19-derived miR-675 regulates bladder cancer proliferation. Thus, in this study, we evaluated the possible regulatory role of H19-derived miR-675 in bladder cancer cell proliferation. We demonstrated that miR-675 expression level was remarkably increased in patients with bladder cancer and bladder cancer cell lines. Furthermore, overexpression of miR-675 markedly increased bladder cancer cell proliferation, whereas miR-675 inhibitor treatment promoted apoptosis and induced G1 phase cell cycle arrest in human bladder cancer cells. Molecular analysis further confirmed that miR-675 downregulated p53 expression, and may thus inhibited p53-mediated cell cycle arrest and apoptosis.

## Materials and methods

### Tissue samples and cell lines

Human bladder tissues were obtained with informed consent from Jiangsu Province Official Hospital, First Affiliated Hospital of Nanjing Medical University. The protocols used in the study were approved by the Hospital’s Protection of Human Subjects Committee. Forty-eight pathologically diagnosed biopsy specimens and adjacent normal tissues were acquired from patients with bladder cancers. The patients’ characteristics are detailed in Table 1. Five cases of normal bladder tissues were obtained from patients without bladder cancer (including patients with benign prostatic hyperplasia). Normal urothelial cells were collected as previously described [24, 25]. Human bladder cancer cells (RT4, HT-1376, 5637, 253J, TCCSUP, T24, and J82) were obtained and maintained as recommended by American Type Culture Collection (ATCC, Manassas, VA).

**Table 1:** The characteristics of patients with bladder cancer

Characteristic	Cases (%)	miR-675^a^	*p* value
Total	48	3.87 ± 1.52	
Gender			0.2608
Male	33 (68.8)	4.05 ± 1.53	
Female	15 (31.2)	3.51 ± 1.47	
Mean age (years, range)	65 (43-77)		
T stage			<0.0001
Ta	3 (6.3)	0.71 ± 0.16	
T1	13 (27.1)	2.91 ± 1.26	
T2	16 (33.3)	3.99 ± 0.63	
T3	12 (25.0)	4.78 ± 0.76	
T4	4 (8.3)	6.28 ± 0.69	
N stage			<0.0001
N0N1	35 (72.9)	3.39 ± 1.39	
Higher	13 (27.1)	5.21 ± 0.98	
Grade			<0.0001
1–2	8 (16.7)	1.59 ± 1.26	
3	28 (58.3)	3.93 ± 0.89	
4	12 (25.0)	5.28 ± 0.97	

^a^Mean ± SD, relative expression of miR-675 in bladder cancer tissues compared with adjacent normal tissues

### Quantitative real-time PCR

Total RNA was extracted from cancer tissues or cells using Trizol reagent (Life Technologies, Carlsbad, CA) according to the manufacturer’s protocol. For miR-675 detection, reverse transcription (RT) was conducted with Applied Biosystems™ TaqMan® MicroRNA Reverse Transcription Kit (Life Technologies, Carlsbad, CA). The primers for the miR-675 were purchased from Life Technologies (4427975, Carlsbad, CA). U6 was used for normalization. H19 expression was performed as Yan et al. described [22]. The ABI StepOne Plus (Applied Biosystems, Foster City, CA) was used to perform the amplification reaction. And, the date was analyzed by the 2^−∆∆Ct^ method. Each experiment was performed in triplicate.

### Transfection

To overexpress H19, H19 cDNA (GenBank accession no. NR_002196.1) was constructed into the multiple cloning sites of pcDNA3.1 vector (Invitrogen) according to Zhuang et al. [18, 9]. Knockdown expression of H19 was performed by transfection with specific siRNA or its negative control (H19: 4390771, Life technologies, Carlsbad, CA), respectively. A total of 0.5 μg of empty vector or pcDNA-H19 was transfected into bladder cancer cells by using Lipofectamine 2000 (Invitrogen™, Life Technologies), respectively. miR-675 mimic, inhibitor, and negative control were purchased from Life Technologies (Carlsbad, CA). A total of 2 × 10^5^ cells were plated in 24-well plate for 24 h and then transfected with 15 nM of miR-675 mimic/inhibitor/negative control by using lipofectamine 2000 (Invitrogen™, Life Technologies) for 48 h. The cells were then subjected to RNA/protein extraction or further functional assays.

### Cell proliferation

Cell proliferation assays were carried out with a Cell Counting Kit-8 (Beyotime, shanghai, China) [26]. 253J and RT4 bladder cancer cells were plated in 24-well plates at approximately 2 × 10^5^ cells per well. Cells were then transfected with pcDNA-H19 or H19-siRNA, and the numbers of cells per well were detected by the absorbance (450 nm) of reduced WST-8 at the indicated time points. The absorbance (450 nm) was measured by using SpectraMax® i3x microplate reader (Molecular Devices, Sunnyvale, CA).

### Cell apoptosis

Evaluation of bladder cancer cell apoptosis was performed by using FITC Annexin V Apoptosis Detection Kit with PI (Biolegend, San Diego, CA). The cells were washed twice with cold BioLegend’s Cell Staining Buffer, and then resuspended cells in Annexin V Binding Buffer at a concentration of 0.25–1.0 × 10^7^ cells/ml. This suspension (100 μl) was stained with 5 μl of FITC Annexin V and 10 μl of propidium iodide. Then, gently vortexed the cells and incubated for 15 min at room temperature (25 °C) in the dark. Added 400 μl of Annexin V Binding Buffer to each tube and analyzed by flow cytometry.

### Cell cycle analysis

Bladder cancer cells were trypsinized, fixed in 70 % ethanol at least 30 min at 4 °C. Then, washed with phosphate-buffered saline (PBS) and incubated in 200 μl PBS containing PI (50 μg/ml) and RNAse A (20 μg/ml, Sigma) for 30 min at 37 °C. Samples were then analyzed for their DNA content by BD accuri™ C6.

### Western blot

Proteins were extracted from cells with RIPA lysis buffer (Beyotime, China) and were quantified using a BCA Protein Assay Kit (Beyotime, China). Whole cell lysates (40 μg of protein lysates for detecting p53, p21, cyclinD1 and β-actin, and 80 μg for detecting Bcl2, Bax) were run on 8 or 12 % SDS-PAGE and then transferred to PVDF membranes (Millipore, USA). The membrane was blocked in 5 % nonfat milk and incubated overnight with the specific primary antibody at 4 °C, and further incubated for 1 h with the HRP-conjugated secondary antibody. The immunoreactive bands were visualized by Immobilon™ Western Chemiluminescent HRP Substrate (ECL) (Millipore, USA), using UVP Bioimaging system (UVP, Upland, CA).

### Statistical analysis

All data are expressed as means ± standard deviation (SD) from at least three separate experiments and analyzed by Graphpad Prism V.5.00 software (San Diego, CA, USA). The differences between groups were evaluated by using Student’s *t* test (two-sided) or one-way ANOVA. *p* < 0.05 was considered statistically significant.

## Results

### miR-675 expression is significantly upregulated in bladder cancer

**Fig. 1:**
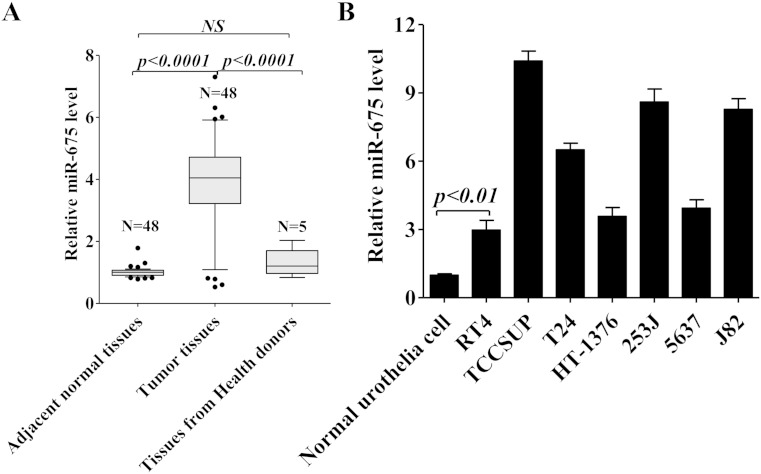
miR-675 levels are upregulated in bladder cancer. **a** miR-675 expression in bladder cancer tissues, adjacent normal control tissues (*n* = 48) and tissues from health donors (*n* = 5) were analyzed by qRT-PCR. Total RNA was extracted and subjected to qRT-PCR to analyze the CT values of bladder cancer normalized to U6 in each sample. The normalized value (∆Ct) of bladder cancer tissue or tissue from health donor was then compared with normal tissue in each group (∆∆Ct). The results are expressed as 2^−∆∆Ct^. **b** miR-675 levels were evaluated by qRT-PCR in seven bladder cancer cell lines. Normal urothelial cells were used as control. Data are presented as mean ± SD (*n* = 3)

H19 has been shown to promote the proliferation and metastasis of bladder cancer [9, 10], as the derivative of H19, miR-675 also abnormally enhanced cancer cell proliferation, like gastric cancer, glioma, and colorectal cancer [22, 17, 18]. In order to further identify whether miR-675 induces bladder cancer proliferation, we first examined the expression of miR-675 in bladder cancer tissues, adjacent normal control tissues, and normal bladder tissues from patient without bladder cancer. As shown in Fig. 1a, the expression of miR-675 was significantly upregulated in most bladder cancer tissues; however, this increase was not observed in patients without bladder cancer. Furthermore, increased expression of miR-675 was also observed in several human bladder cancer cell lines (RT4, HT-1376, 5637, 253J, TCCSUP, T24, and J82) (Fig. 1b). Thus, these data indicate that upregulation of miR-675 may be related to the progression of bladder cancer.

### miR-675 promotes bladder cell proliferation in vitro

**Fig. 2:**
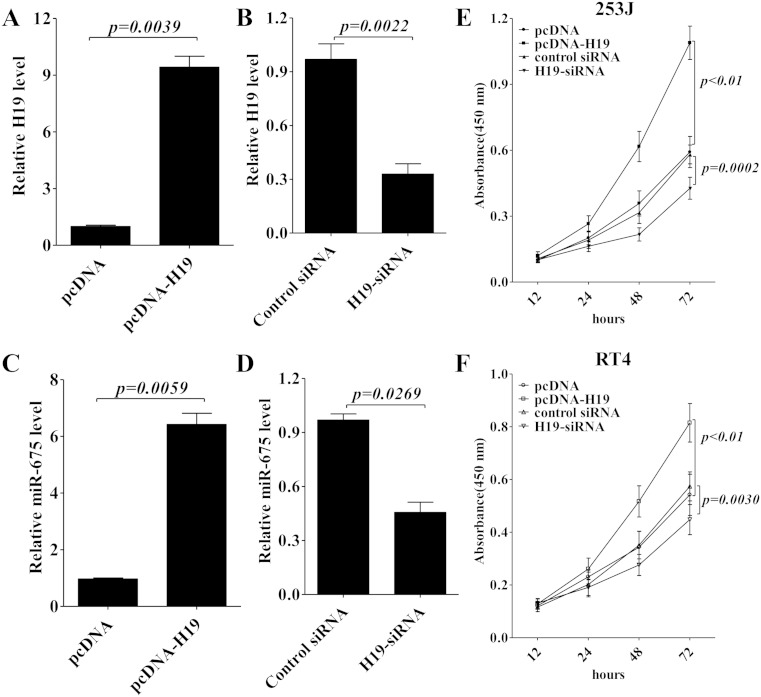
H19 promotes cell proliferation and miR-675 expression in bladder cancer cells. 253J cells were transiently transfected with pcDNA-H19 or H19-siRNA. Forty-eight hours later, the H19 (**a**, **b**) and miR-675 (**c**, **d**) expression in H19 knockdown or overexpressed cells was validated using qRT-PCR. Cell proliferation of 253J cells (**e**) and RT4 cells (**f**) were evaluated by the absorbance (450 nm) of reduced WST-8. The absorbance at 450 nm was measured at 12, 24, 48, and 72 h after transfection. Data are presented as mean ± SD (*n* = 3)

As a mature product of H19, miR-675 is the pivotal intermediator that H19 exploited to enhance the carcinogenesis and metastasis of different cancers [27, 17, 23]; so, we further examined the regulatory role of H19 in miR-675 expression in 253J and RT4 bladder cancer cells. First, the expression of H19 was interfered or overexpressed in 253J cells, as shown in Fig. 2a; ectopic expression of H19 (Fig. 2a) caused a significant upregulation of miR-675 expression (Fig. 2c) and increased cell proliferation of 253J cells and RT4 cells (Fig. 2e, f). Furthermore, H19-siRNA treatment (Fig. 2b) decreased the miR-675 expression level (Fig. 2d) and inhibited 253J cell proliferation (Fig. 2e, f). So, these data suggest that H19 promotes cell proliferation and miR-675 expression in bladder cancer.

**Fig. 3:**
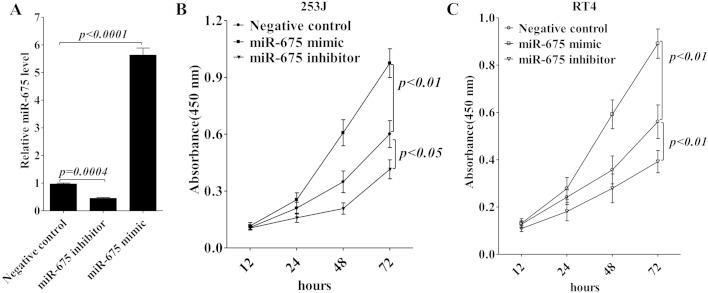
miR-675 increases bladder cancer cell proliferation. **a** 253J cells were transiently transfected with miR-675 mimic or inhibitor. Forty-eight hours later, the miR-675 expression was validated using real-time RT-PCR. Cell proliferation of 253J cells (**b**) and RT4 cells (**c**) was evaluated by the absorbance (450 nm) of reduced WST-8. The absorbance at 450 nm was measured at 12, 24, 48, and 72 h after transfection. Data are presented as mean ± SD (*n* = 3)

As H19 has been shown to promote proliferation and metastasis of bladder cancer [9, 10], then we treated 253J cells with miR-675 mimic or inhibitor to verify the role of miR-675 in regulating proliferation of 253J cells and RT4 cells. miR-675 expression was significantly enhanced or decreased with the transfection of microRNA into 253J cells (Fig. 3a), and more importantly, overexpression of miR-675 enhanced cell proliferation of 253J cells (Fig. 3b) and RT4 cells (Fig. 3c), while miR-675 inhibitor showed a significant inhibitory role in cell proliferation. Thus, these data confirm that upregulation of miR-675 in bladder cancer increases tumor cell growth in vitro.

### miR-675 promotes cell cycle progression and inhibits cell cycle arrest

**Fig. 4:**
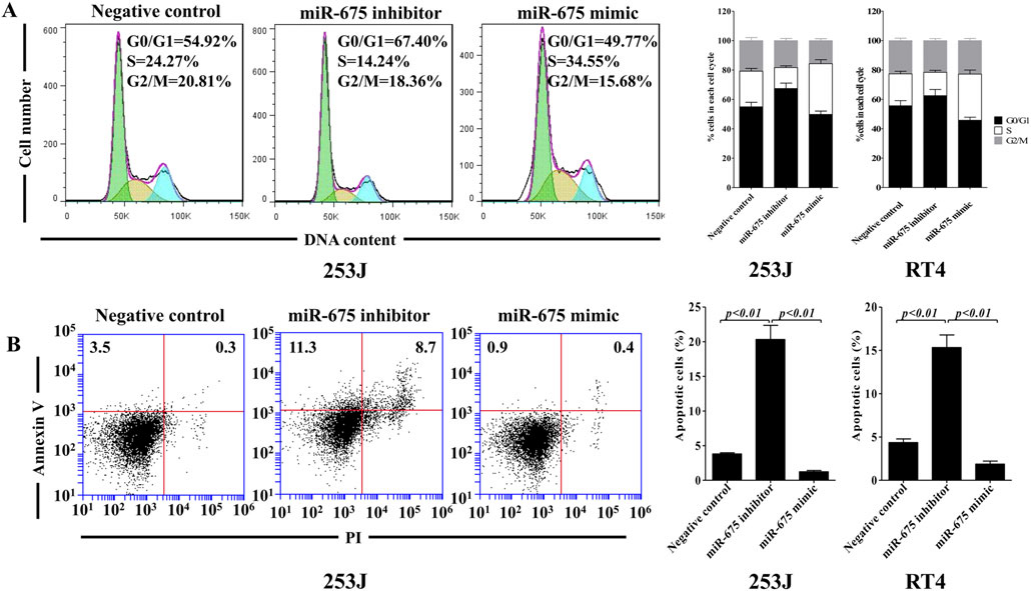
Downregulation of miR-675 expression induces bladder cancer cell apoptosis and G1 phase cell cycle arrest. 253J cells and RT4 cells were transiently transfected with miR-675 mimic or inhibitor. Forty-eight hours later, cell cycle arrest (**a**) and apoptosis (**b**) were analyzed by flow cytometry. Data are presented as mean ± SD (*n* = 3)

To further evaluate whether this miR-675-induced promotion of cell proliferation was due to inhibit cell cycle arrest and/or apoptotic death, we first examined the effect of miR-675 on cell cycle of 253J cells and RT4 cells. As shown in Fig. 4a, cells in the S phase were significantly increased, and cells in the G0/G1 phase were significantly decreased by the miR-675 mimic treatment in both these two cell lines. Next, we assessed whether downregulated expression of miR-675 contributes to cell apoptosis. Figure 4b shows that miR-675 inhibitor significantly promoted the apoptosis of bladder cancer cells. Taken together, these data suggest that miR-675 positively regulates the proliferation of bladder cancer cells through cell cycle arrest and apoptosis inhibition.

### miR-675 suppresses p53 activation

**Fig. 5:**
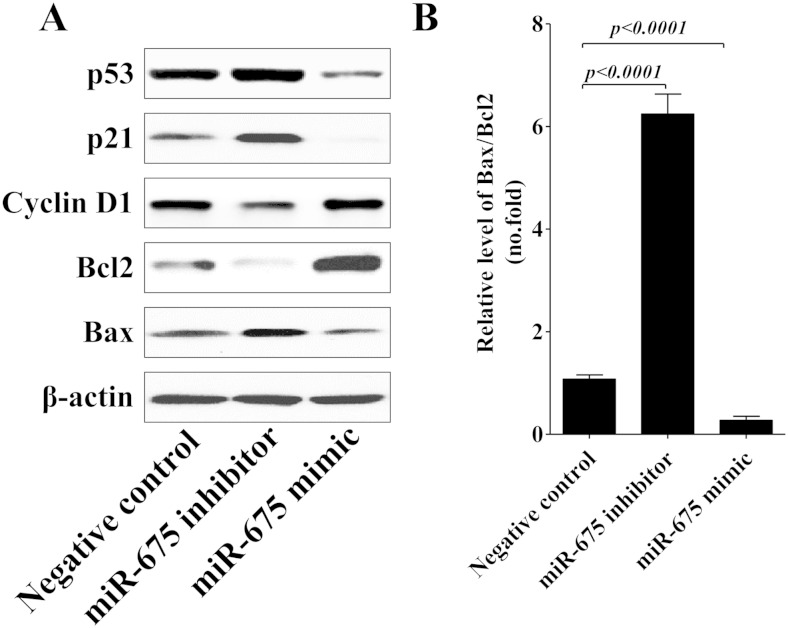
miR-675 inhibits p53 and p53-dependent protein expression. 253J cells were transiently transfected with miR-675 mimic or inhibitor. Forty-eight hours later, whole cell lysate was used for the Western blotting analysis. p53, p21, cyclin D1, Bcl-2, Bax, and β-actin were detected with their respective antibodies (**a**). Relative expression of Bax and Bcl-2 was analyzed by *gray scale* (**b**), β-actin was used as endogenous control. Data are presented as mean ± SD (*n* = 3)

Compared with miR-675 mimic, miR-675 inhibitor significantly induced G0/G1 arrest in 253J cells (Fig. 4a), and more importantly, it has been well established that tumor suppressor p53 induces G1 arrest in cells in response to stress [28, 29]. The major downstream effectors of p53 include p21 and cyclin D1, which mediate G(1)-S phase cell cycle progression [30, 31]. So, we further detected the expression of p53 in response to miR-675 treatment. As shown in Fig. 5a, miR-675 significantly decreased p53 expression, as well as p21; furthermore, miR-675 enhanced cyclin D1 expression. These results indicate that miR-675 may promote cell cycle progression by reducing p53 expression, and thus inhibiting p53-mediated cell cycle arrest.

Besides p21 and cyclin D1, p53 could also regulate a variety of target genes involved in apoptosis including Bcl-2 and Bax [32]. Increase in the ratio of Bax/Bcl-2 is known to initiate apoptosis, in Fig. 5a, b, miR-675 mimic markedly downregulated the expression ratio of Bax/Bcl-2, and miR-675 inhibitor significantly enhanced the expression ratio of Bax/Bcl-2. Therefore, these data hint that upregulation of miR-675 expression may inhibit cell apoptosis by regulating p53 activation in bladder cancer.

## Discussion

Currently, emerging evidence has confirmed that the human long noncoding RNA H19 is upregulated in many cancers and promotes cancer progression, including bladder cancer [13, 27, 22, 17, 9, 10, 33]. The mechanisms involved in the regulatory role of H19 are complex, and mostly associated with its function as the precursor of miR-675 [22, 17, 23, 18, 27]. However, whether miR-675 involved in H19-mediated progression of bladder cancer remains unclear. In the present study, we investigated the involvement of H19-derived miR-675 and p53 in cell proliferation of bladder cancer. Our results demonstrated that miR-675 increases bladder cancer cell proliferation by inhibiting cell cycle arrest and apoptosis, and this effect may be depending on its downregulation of p53 expression.

H19 expression is significantly correlated with tumor grade and is a marker of early recurrence in bladder cancer [13, 12, 9, 10]. As the mature product of H19, our data showed that miR-675 was expressed at higher levels in bladder cancer tissues than in adjacent noncancerous tissues. H19 positively regulated miR-675 expression in bladder cancer cells, and upregulation of miR-675 significantly promoted cell proliferation of bladder cancer cells in vitro. To further identify whether miR-675-induced cell proliferation was due to cell cycle arrest and/or enhanced apoptosis, 253J and RT4 bladder cancer cells were transfected with miR-675 mimic or inhibitor and analyzed by flow cytometry. Our data showed that miR-675 overexpression in cells significantly reduced G1 arrest and cell apoptosis. It has been well established that p53 induces G1 arrest in tumor cells; we thus hypothesize that p53 may negatively regulate miR-675 in bladder cancer cells.

Many targets of miR-675 have been proposed in different tumors, such as Twist1 and RB in hepatocellular carcinoma and colorectal cancer [34, 17], CALN1 and RUNX1 in gastric cancer [27, 18], TGFBI in prostate cancer [23]. It should be noted that RUNX1 is an important upstream activator of p53 and enhances p53 activity in response to DNA damage in tumor cells [19]. In our chosen cell lines, RT4 and 253J were p53 wild type [35], and the others were p53 mutant [36, 37]. In our results, we found that miR-675 negative regulated the expression of p53 and its downstream proteins in both 253J cells and RT4 cells. However, we found that the effects of miR-675 in RT4 cells were not apparent as in 253J cells; this may be owned to that 253J was invasive bladder cancer cells, while RT4 was noninvasive cells [10]; the expressions of miR-675 and H19 were remarkably lower in RT4 cells. Zhuang et al. demonstrated that miR-675 inhibited proliferation of both p53 wild type and mutant gastric cancer cell lines [18]. In preliminary studies, we have found that miR-675 induced a G2-M arrest in p53 mutant bladder cancer cells (data not shown), and next, we will examine the role of miR-675 involved in proliferation of p53 mutant bladder cancer cells and explore the detailed molecular mechanisms.

In summary, we demonstrated that miR-675 expression is markedly increased in bladder cancer tissues and cell lines compared with normal control. miR-675 was positively regulated by H19, and overexpression of miR-675 increases cell proliferation of human bladder cancer cells. We further confirmed that miR-675 induces a G1 arrest and inhibits cell apoptosis, and this may depend on its negative regulatory role for p53 expression. In conclusion, our results suggest an important role for miR-675 in the molecular etiology of bladder cancer and highlight potential application of miR-675 in bladder cancer therapy.
